# Network pharmacology for systematic understanding of Schisandrin B reduces the epithelial cells injury of colitis through regulating pyroptosis by AMPK/Nrf2/NLRP3 inflammasome

**DOI:** 10.18632/aging.203611

**Published:** 2021-10-09

**Authors:** Weiwei Zhang, Wusan Wang, Chaozhuang Shen, Xiaohu Wang, Zhichen Pu, Qin Yin

**Affiliations:** 1 Department of Pharmacy, Second Affiliated Hospital of Wannan Medical College, Wuhu 241001, Anhui, China; 2 Department of Pharmacology, Wannan Medical College, Wuhu 241001, Anhui, China; 3 Drug Clinical Evaluation, Yijishan Hospital of Wannan Medical College, Wuhu 241001, Anhui, China; 4 Wannan Medical College, Wuhu 241001, Anhui, China

**Keywords:** Schizandrin B, colitis, AMPK, mitochondrial damage, network pharmacology

## Abstract

Ulcerative colitis (UC) is a chronic inflammatory disease with increasing incidence and prevalence in many countries. The purpose of this study is to explore the function of Schisandrin B and its underlying molecular mechanisms in colitis.

In this study, mice with colitis were induced by giving 2.0% dextran sulfate sodium (DSS, MP) in the drinking water for seven days. Furthermore, TCMSP server and GEO DataSets were used to analyze the mechanism of Schisandrin B in colitis.

It was found that Schisandrin B presented colitis in mice model. At the same time, Schisandrin B not only reduced inflammation *in vivo* and *vitro* model of colitis, but also suppressed the nucleotide-binding oligomerization domain, leucine-rich repeat and pyrin domain-containing 3 (NLRP3) inflammasome *in vivo* and *vitro* model of colitis. In addition, Schisandrin B induced AMP-activated protein kinase (AMPK) / Nuclear factor erythroid 2-related factor 2 (Nrf2) signaling pathway in model of colitis, and regulated AMPK protein at 316 sites. The inhibition of AMPK reduced the anti-inflammation effects of Schisandrin B on NLRP3 inflammasome. Apart from that, Schisandrin B decreased reactive oxygen species (ROS)-induced mitochondrial damage and reduced epithelial cells damage of colitis through regulating pyroptosis.

Collectively, our novel findings for first time showed that, Schisandrin B suppressed NLRP3 inflammasome activation-mediated interleukin-1beta (IL-1β) level and pyroptosis in intestinal epithelial cells of colitis model through the activation of AMPK/Nrf2 dependent signaling-ROS-induced mitochondrial damage, which may be a significant therapeutic approach in the treatment of acute colitis.

## INTRODUCTION

Ulcerative colitis (UC) is the inflammation of the rectal mucosa that usually extends proximally to other areas of the colon [1]. UC is characterized by cytokine imbalance, immune dysfunction and intestinal mucosa-associated inflammation [1]. UC patients generally present with bloody diarrhea, abdominal pain and tenesmus, with increasing global incidence [2]. There are approximately 286 cases per 100,000 people in the United States and 505 cases per 100,000 people in Europe [3]. There are limited data in developing countries. However, the incidence of UC is also increasing year by year in Asia, Middle East and South America [4].

The pathogenesis of UC remains unclear at present, which is considered to be associated with the interaction of multiple environmental, genetic and immune factors [5]. The common pathogenic feature is the destruction of the integrity of the intestinal epithelial barrier. Under normal circumstances, several types of cellular proteins secreted by epithelial cells can support the intestinal mucosal barrier. The barrier is mainly composed of intercellular junction complexes, consisting of tight junction (TJ) protein (occlusion proteins and claudin-1) and zona [6]. The destruction of TJ leads to the disorder of the paracellular barrier and the increasing permeability of the intestinal epithelial cells [7]. This change in permeability can cause the penetration of harmful antigens and intraluminal bacteria into the intestine, leading to the initiation and acceleration of mucosal inflammation of UC [8]. Therefore, therapies to attenuate intestinal barrier dysfunction can effectively treat UC.

AMP-activated protein kinase (AMPK) is a highly conserved cellular energy sensor. Under inflammatory pathological conditions, the phosphorylation level of AMPK is decreased, which subsequently affects the expression of inflammatory mediators and downstream biological functions [9, 10]. AMPK activators can exert anti-inflammatory effects [11, 12].

AMPK may inhibit the pathogenesis of inflammatory bowel disease (IBD) by inhibiting the activity of nuclear factor-kappaB (NF-kappaB) [13]. The expression of NF-κB is increased in the colon lesion of IBD patients [14]. In the dextran sulfate sodium (DSS)-induced mouse colitis model, metformin, an AMPK activator, could significantly inhibit the activation of IKK induced by DSS, thereby down-regulating the expression of the inflammatory factor IL-18 [15]. Additionally, AMPK can suppress the activation of NLRP3 inflammasome by decreasing NF-κB activation, thereby attenuating colitis [16].

Pyroptosis is a form of intestinal cell death, which is characterized by swollen cells and the appearance of large bubbles on the cell membrane [17]. Studies have reported that attenuating pyroptosis can relieve experimental colitis [17, 18]. Studies have also shown that the nucleotide-binding oligomerization domain, leucine-rich repeat and pyrin domain-containing 3 (NLRP3) inflammasome is an important signal transduction pathway of colitis injury, playing an important role in regulating cell proliferation, differentiation and apoptosis [18, 19]. Caspase-1 and NLRP3 are key components of inflammation, and their dysregulation promotes the production of UC-associated pro-inflammatory cytokines (interleukin-1beta (IL-1β) and IL-18) [20].

Inflammasome proteins include NLRP1, NLRP3, NLRC4, and NLRP6, etc., which are assembled with apoptosis-associated speck-like protein (ASC) and pro-inflammatory caspases (Caspase-1 and Caspase-11) into the inflammasome complex [21]. It can not only lead to the automatic activation of caspases and control the cleavage of IL-1β and IL-18 precursors into their mature forms, but also induce pyroptosis [22]. The IL-1 cytokine family can regulate the intestinal homeostasis, inflammation and healthy microbiota [23]. To be specific, NLRP3 inflammasome induces IL-1β in myeloid cells and promotes intestinal inflammation [23]. Therefore, the function of NLRP3 in mucosal immunity and colitis seems to be complex, with unclear underlying mechanism [24].

Natural products commonly used in traditional Chinese medicine (TCM) have attracted accumulative medical interest globally due to their powerful anti-inflammatory effects [25]. Schisandra chinensis, a type of TCM, is originally produced in northeastern China, South Korea, eastern Russia and Japan, which is used to treat various diseases, including autoimmune diseases, cardiovascular diseases, acne and neurological diseases [26]. At present, the chemical composition of Schisandra chinensis is not completely understood. Schisandrin B is the most important ingredient in Schisandra chinensis, which has been revealed to have anti-cancer, anti-inflammation, hepatoprotection, anti-bacteria and many other activities by accumulative evidence [27]. Schisandrin B and Schisandrol are representative lignans of Schisandra chinensis [27]. Multiple studies have demonstrated their anti-inflammatory effects. According to relevant reports, Schisandrin B can significantly inhibit the level of inflammation by blocking NLRP3 activation and of IL-1β secretion [28]. However, there is no study concerning the specific mechanism of Schisandrin B in attenuating pyroptosis. Meanwhile, there is also no study on Schisandrin B in relieving sepsis-induced colitis by regulating pyroptosis [29, 30]. Additionally, Schisandrin B has also been revealed to play a protective role in arthritis [31]. Therefore, in this study, we mainly investigate the molecular mechanism of Schisandrin B in relieving epithelial cell injury and regulating pyroptosis in colitis.

## MATERIALS AND METHODS

### Animal care model of colitis and treatment

All experiments related to animal were approved by the Animal Care and Use Committee of Wannan Medical College. All C57BL/6 mice (5-6 weeks, 18-20 g) were obtained from Animal testing center of Qinglongshan (Nanjing, Suzhou, China). C57BL/6 mice were randomly assigned to three groups: sham group (n = 6), model group (n = 8), 10 mg/kg Schisandrin B (Sigma-Aldrich LLC.) treatment group (n = 8). All mice of model group were induced by giving 2.0% dextran sulfate sodium (DSS, MP) in the drinking water for 7 days. All mice of Schisandrin B were given with 10 mg/kg/ per day Schisandrin B body weight by intraperitoneal injection 1 h, and then induced by giving 2.0% dextran sulfate sodium (DSS, MP) in the drinking water for 7 days. Weight of every mice were measured at every day.

AMPK i group (AMPK inhibitor, n = 8), all mice of Schisandrin B were given with 10 mg/kg/day Schisandrin B (7 day) and 10 mg/kg of Dorsomorphin (7 day, MedChemExpress, i.p.) body weight by intraperitoneal injection 1 h, and then induced by giving 2.0% dextran sulfate sodium (DSS, MP) in the drinking water for 7 days.

After induction model, mice were anaesthetized with isoflurane (induction: 3-5%, maintenance: 1.5-3%), and peripheral blood were collected form submaxillary vein. Then, mice with sacrificed using cervical dislocation under anaesthesia (1.5-3% of isoflurane). Colon (1 cm) was collected and fixed in 4% paraformaldehyde for histological examination. colon was collected and saved at -80° C for other experiment.

Assessment of disease activity index (DAI): body weight loss <2%, 0 Score; body weight loss≥2%-<5%, 1 Score; body weight loss≥5%-<10%, 2 Score; body weight loss≥10%-<15%, 3 Score; body weight loss≥15%, 4 Score.

### Bioinformatics analysis

GEO DataSets and TCMSP server (http://ibts.hkbu.edu.hk/LSP/tcmsp.php) were a systems-level pharmacology database and a flexible, user-friendly web interface. Targets gene and diseases, networks were constructed and analyzed using Cytoscape 3.0 for a deeper understanding of the complex.

### Histological examination

Colon tissue samples were fixed in 4% paraformaldehyde, paraffin-embedded and then sectioned into 5 μM slices for H&E staining. Colon tissue samples were observed using fluorescence microscope (Zeiss Axio Observer A1, Germany). Colon tissue scored according to the following criteria: 1, normal; 2, minimal (little) damage; 3, mild damage; 4, moderate damage; 5, severe damage; and 6, maximal damage as document [13].

### Cell culture and treatment

HCT-116 cells were seeded in culture dish with RPMI 1640 (Gibco) supplemented with 10% FBS (Gibco) under a humidified 5% (v/v) CO2 atmosphere at 37° C. HCT-116 cells of vitro model group were stimulated with 500 ng/ml of LPS (Sigma-Aldrich, MO, USA) for 4 h, and then pulsed with 1 mM of ATP (Sigma-Aldrich, MO, USA) for 30 min as references [32].

HCT-116 cells of Schisandrin B group were stimulated with 500 ng/ml of LPS (Sigma-Aldrich, MO, USA) or 40 μM of Schisandrin B for 4 h without FBS, and then pulsed with 1 mM of ATP (Sigma-Aldrich, MO, USA) for 30 min.

AMPK i group of HCT-116 cells were stimulated with (500 ng/ml) LPS+40 μM of Schisandrin B + epiberberine (25 μM, MedChemExpress) for 4 h, and then pulsed with 1 mM of ATP (Sigma-Aldrich, MO, USA) for 30 min as document [33].

### ELISA assay, ROS production and JC-1 assay

After induction model, mice were anaesthetized with Isoflurane (Induction: 3-5%, Maintenance: 1.5-3%), and peripheral blood were collected form submaxillary vein. Blood was centrifugated at1000 g at 4° C for 10 min and serum was collected and saved at -80° C for other experiment. Blood or cell samples were collected and used to measure ROS (E004-1-1), TNF-α (H052-1), IL-6 (H007-1-1), IL-18 (H015) and IL-1β (H002) levels using ROS, TNF-α, IL-6, IL-18 and IL-1β ELISA kits (Nanjing Jiancheng Biological Engineering Institute, Nanjing, China) following the manufacturer’s instructions.

1x 105/well cells were seeded into a 96-well plate and 100 μL JC-1 probe solution (C2006, Beyotime Biotechnology) was added into every well as previously described [34]. Absorbance was measured using a fluorescent reader (Synergy H1 Microplate Reader, Bio Tek, VT, USA).

### Western blotting analysis

Colon tissue samples or cell samples or supernatant samples were split using RIPA assay (Beyotime) in ice. Total proteins were quantified using BCA assay (Beyotime) and were electrophoresed on 10% SDS-acrylamide gels. Total proteins were transferred to nitrocellulose membranes and membranes were blocked with 5% non-fat milk in TBS for 1 h at 37° C. Membranes were incubated with p-AMPK (ab23875, abcam), AMPK (ab32047, abcam), Nrf2 (ab62352, 1:1000, abcam), GSDMD (ab219800, 1:1000, abcam), NLRP3 (sc-66846, 1:500, Santa Cruz, USA), caspase-1 (sc-1780, 1:500, Santa Cruz, USA), and β-Actin (BS6007MH, 1:5000, Bioworld Technology, Inc.) at 4° C overnight. The membranes were incubated with horseradish peroxidase-conjugated secondary antibodies (sc-2004 or sc-2005, 1:5000, Santa Cruz, USA) for 1 h at 37° C after washing with TBST for 15 min. Protein was measured using an enhanced chemiluminescence system (ECL, Beyotime) and analyzed using an Image Lab 3.0 (Bio-Rad Laboratories, Inc.).

### Immunofluorescence

Cell were washed with PBS and fixed with 4% paraformaldehyde supplemented with 0.25% Tris-X100 at room temperature for 10 min. After blocking with PBS supplemented with 5% BSA for 2 h at room temperature, cells were incubated with Mito-Tracker Red CMXRos (C1035, Beyotime), NLRP3 (sc-66846, 1:500, Santa Cruz, USA), at 4° C overnight. Cells were incubated with secondary peroxidase conjugated goat anti-rabbit IgG (1:100, Santa Cruz Biotechnology) antibody for 2 h at room temperature, after washing with PBST for 15 min. Cells were stained with DAPI for 15 min at darkness, after washing with PBST for 15 min. Cell samples were observed using fluorescence microscope (Zeiss Axio Observer A1, Germany).

Next, cells were washed with PBS and stained with Mito-Tracker Red CMXRos for 30 min. Cells washed with PBST for 15 min and stained with DCFH-DA for 30 min. Cell samples were observed using fluorescence microscope (Zeiss Axio Observer A1, Germany) after washing with PBST for 15 min.

Cell were washed with PBS and fixed with 4% paraformaldehyde for 15 min. Cells were stained with PI for 15 min at darkness, after washing with PBST for 15 min. Cell samples were observed using fluorescence microscope (Zeiss Axio Observer A1, Germany).

### Electron microscopy

Cells were prepared as previously document [35]. 1 x 10^6^ HCT-116 cells were fixed in 0.2 M phosphate buffer supplemented with 2.5% glutaraldehyde (G5882, Sigma-Aldrich) for 4 h. Cells were post-fixed for 60 min in 1% osmium tetroxide (75632, Sigma-Aldrich) and dehydrated in gradient ethanol solutions (15 min each step). Cells were infiltrated sequentially in ethanol and 1:3 of ethanol for 30 min, and finally 100% spurr resin for 24 h at 60° C for polymerization. Cells were examined at 80 kV using a Hitachi H7650 transmission electron microscope (Tokyo, Japan).

### Structure the protein of AMPK and molecular docking verification

Molecular docking was performed between the screened representative components and the target gene. The “PDB” file of the protein skeleton of the target gene was downloaded using RCSB PDB database, and the “mol2” file of the molecular structure diagram of the representative components was downloaded using tcmsp database. Import the target gene protein skeleton file into Python PyMOL 2.5.0 software, and use the “remove solvent” and “remove organic” commands to remove water and ligands. Import the processed two types of files into autodock tools software to create a “pdbqt” file, then use autodock Vina software for molecular docking and calculate the binding energy of each conformation, select the conformation with the lowest binding energy to dock with the target protein, save the docked complex file, and import the complex file into Python PyMOL 2.5.0 software for final visualization and local optimization.

This experiment used molecular biology to reform the protein of AMPK, 316-Serine changed 316-Glu.

WT (PRKAA1, 5’-CGGATCCTGCGCAGACTCAGTTCCTGGAGGAA-3’ and 5’-AATCTAATTAAAATTCTTGCACAATAAACTATCGATG-3’, Sangon Biotech Co., Ltd.) and Mutant (5’-CAAGCTTAGTTTGAGTGCGAAGAAGAGGAAGTTA-3’ and 5’-CTCAAAGCACTTCTTCTCCTTCAAGGCGGCGGCAA-3’, Sangon Biotech Co., Ltd.) plasmids were transfected into HCT-116 cells using Lipofectamine 2000 for 48 h. Then, HCT-116 cells were stimulated with 500 ng/ml of LPS (Sigma-Aldrich, MO, USA) and 40 μM of Schisandrin B for 4 h, and then pulsed with 1 mM of ATP (Sigma-Aldrich, MO, USA) for 30 min.

### Cell proliferation and LDH activity

HCT-116 cells were induced for vitro model. Then, 100 μl of cell suspension (1×10^3^/ml) was inoculated into a 96-well plate at 37° C. 10 μl CCK-8 was added followed by cultivation in an incubator at 37° C for 1-2 h. The cell proliferation curve was plotted based on optical density (OD) values. OD values were performed on a microplate reader with a detection wavelength of 450 nm.

HCT-116 cells were induced for vitro model. LDH activity levels were measured using LDH activity kit ELISA kits (C0016, Beyotime, Nanjing, China) following the manufacturer’s instructions.

### Flow cytometry

HCT-116 cells were induced for vitro model. Then, cells were collected to measure apoptosis rate using Annexin V-FITC Apoptosis Detection Kit (C1062M Beyotime, Nanjing, China) following the manufacturer’s instructions. Cells were fixed with 4% paraformaldehyde for 15 min and stained with PI and Annexin V-FITC for 15 min at darkness at room temperature. HCT-116 cells were checked using Flow cytometry (C6, BD Biosciences).

### Statistical analysis

Data were expressed as mean ± SEM. Multiple comparisons were used GraphPad Prism 8 to perform by one-way ANOVA followed by Tukey’s post-test or Kruskal-Wallis test followed by Dunn’s post hoc test. P values < 0.05 were considered statistically significant.

### Data availability

If the data are all contained within the manuscript and/or Supporting Information files, enter the following: All relevant data are within the manuscript and its Supporting Information files.

## RESULTS

### Schisandrin B presented colitis in mice model

**Figure 1:**
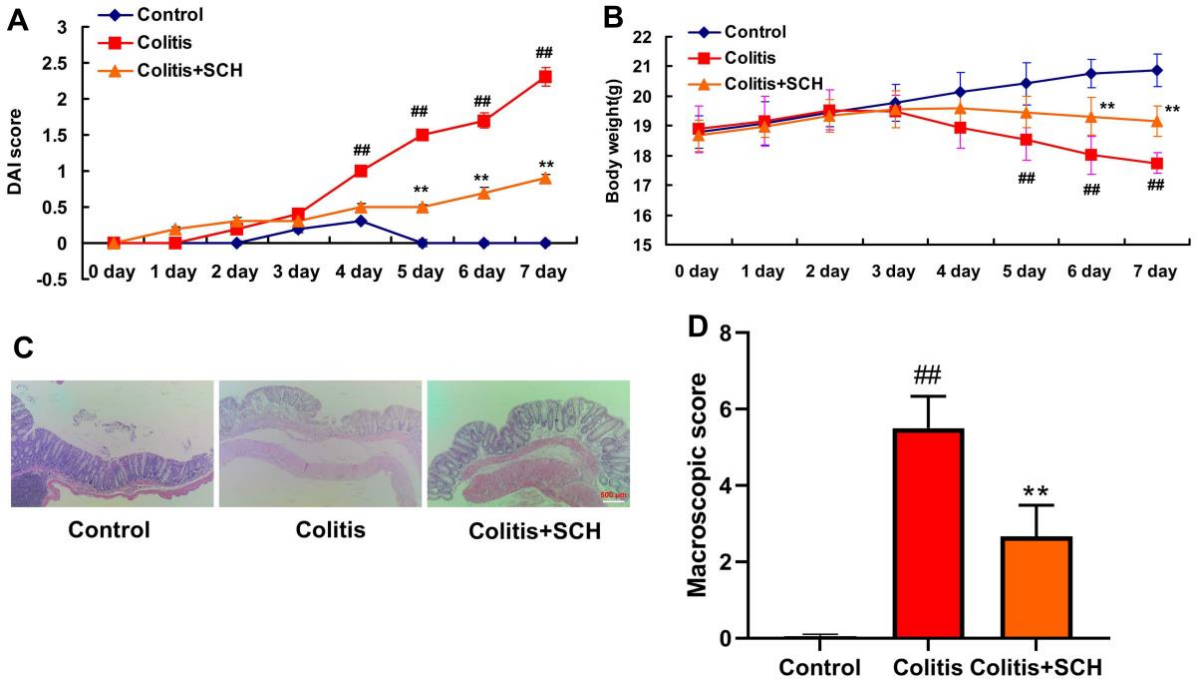
**Schisandrin B presented colitis in mice model.** (**A**) DAI score; (**B**) weight of mice; (**C**) HE staining results of colon (×200); (**D**) Colon macroscopic score. ##P<0.01 vs control group; **P<0.01 vs DSS- induced colitis group. Control: blank control group; Colitis: DSS- induced colitis group; Colitis+SCH: Schisandrin was used to treat DSS- induced colitis. SCH, Schisandrin B. Data were expressed as mean ± SEM.

In this experiment, the effect of Schisandrin B in mice model of colitis was explored. The ulcer area, ulcer score, body weight and DAI score in DSS induced colitis mice model group were significantly increased than those in the control normal group (Figure 1). Schisandrin B could not only reduce the ulcer area and ulcer score, but also recover the weight and DAI score in DSS induced colitis mice. Meanwhile, there were significant differences in comparison to the DSS induced colitis model group (Figure 1).

### Schisandrin B reduced inflammation *in vivo* and *vitro* model of colitis

**Figure 2:**
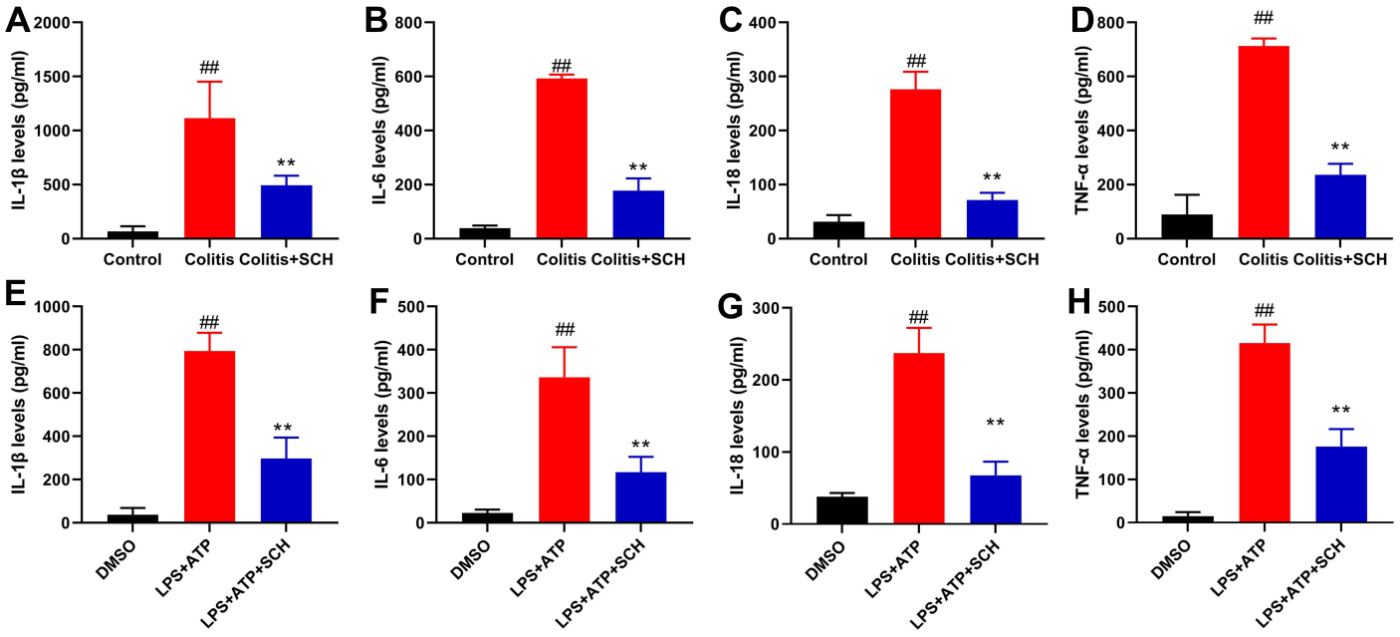
**Schisandrin B reduced inflammation *in vivo* and *vitro* model of colitis.** (**A**–**D**) The levels of TNF-α, IL-6, IL-1β and IL-18 in colon tissue; (**E**–**H**) the levels of TNF-α, IL-6, IL-1β and IL-18 in intestinal epithelial cells. ##P<0.01 vs control group; **P<0.01 vs DSS- induced colitis group. Control: blank control group; Colitis: DSS- induced colitis group; Colitis+SCH: DSS- induced colitis mice with Schisandrin. ##P<0.01 vs MDSO group; **P<0.01 vs LPS+ATP induced intestinal epithelial cells group. MDSO: blank control group; LPS+ATP: intestinal epithelial cells with LPS+ATP group; LPS+ATP +SCH: intestinal epithelial cells induced by LPS+ATP with Schisandrin. SCH, Schisandrin B. Data were expressed as mean ± SEM.

This study determined whether Schisandrin B reduced inflammation *in vivo* and vitro model of colitis. It was found that serum TNF-α, IL-6, IL-18 and IL-1β levels in DSS induced colitis mice model was significantly higher than those in the control normal group (Figure 2A–2D). Compared with the DSS induced colitis mice model group (Figure 2A–2D), administration of Schisandrin B more significantly reduced the serum TNF-α, IL-6, IL-18 and IL-1β levels in DSS induced colitis mice model. Apart from that, LPS+ATP induced the supernatant TNF-α, IL-6, IL-18 and IL-1β levels in intestinal epithelial cells (Figure 2E–2H). Schisandrin B reduced the supernatant TNF-α, IL-6, IL-18 and IL-1β levels in intestinal epithelial cells by LPS+ATP (Figure 2E–2H).

### Schisandrin B suppressed NLRP3 inflammasome *in vivo* and *vitro* model of colitis

**Figure 3:**
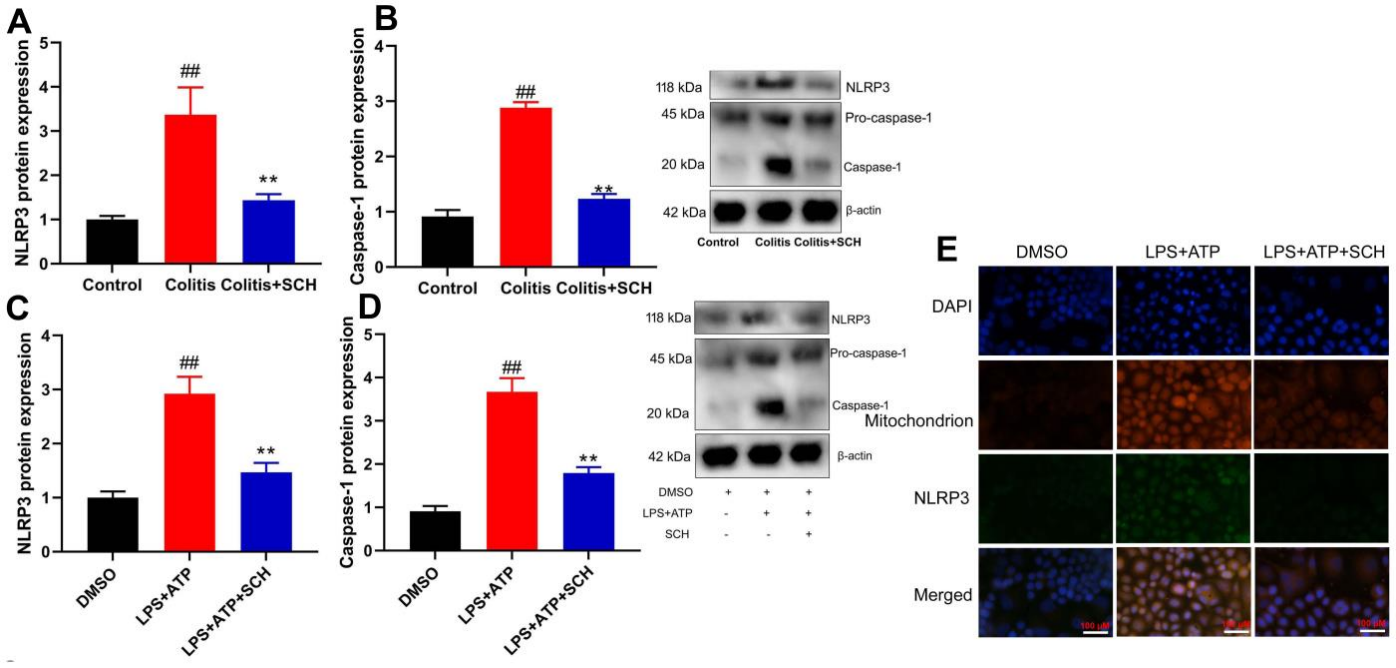
**Schisandrin B suppressed NLRP3 inflammasone *in vivo* and *vitro* model of colitis.** (**A**, **B**) the protein expression of NLRP3 and caspase-1 in mouse colon tissue; (**C**, **D**) the protein expression of NLRP3 and caspase-1 in intestinal epithelial cells induced by LPS + ATP; (**E**) the protein expression of NLRP3 and caspase-1 in intestinal epithelial cells detected by cell immunofluorescence. ##P<0.01 vs control group; **P<0.01 vs DSS- induced colitis group. Control: blank control group; Colitis: DSS- induced colitis group; Colitis+SCH: DSS- induced colitis mice with Schisandrin. ##P<0.01 vs MDSO group; **P<0.01 vs LPS+ATP induced intestinal epithelial cells group. MDSO: blank control group; LPS+ATP: intestinal epithelial cells with LPS+ATP group; LPS+ATP +SCH: intestinal epithelial cells induced by LPS+ATP with Schisandrin. SCH, Schisandrin B. Data were expressed as mean ± SEM.

In order to determine the potential mechanisms by Schisandrin B in model of colitis, NLRP3 inflammasome had been previously defined as the key mediators in inflammation reactions in colitis. Furthermore, NLRP3 protein expression in colon tissue of DSS induced colitis mice model was induced (Figure 3A, 3B). On the one hand, administration of Schisandrin B suppressed NLRP3 protein expression in colon tissue of DSS induced colitis mice model (Figure 3A, 3B). On the other hand, LPS+ATP induced the NLRP3 protein expression in intestinal epithelial cells (Figure 3C, 3D). Schisandrin B reduced NLRP3 protein expression in intestinal epithelial cells by LPS+ATP (Figure 3C, 3D). According to immunofluorescence, LPS+ATP induced NLRP3 expression (green) and activated mitochondrial damage (red) in intestinal epithelial cells. Meanwhile, NLRP3 protein coincided with mitochondrial damage in intestinal epithelial cells (Figure 3E). Schisandrin B suppressed NLRP3 expression (green) and reduced mitochondrial damage (red) in intestinal epithelial cells by LPS+ATP (Figure 3E). These results suggest that Schisandrin B suppressed NLRP3 inflammasome *in vivo* and *vitro* model of colitis, which may be attributed to mitochondrial damage.

### Systematic understanding of how Schisandrin B induced AMPK/Nrf2 signaling pathway in colitis model

**Figure 4:**
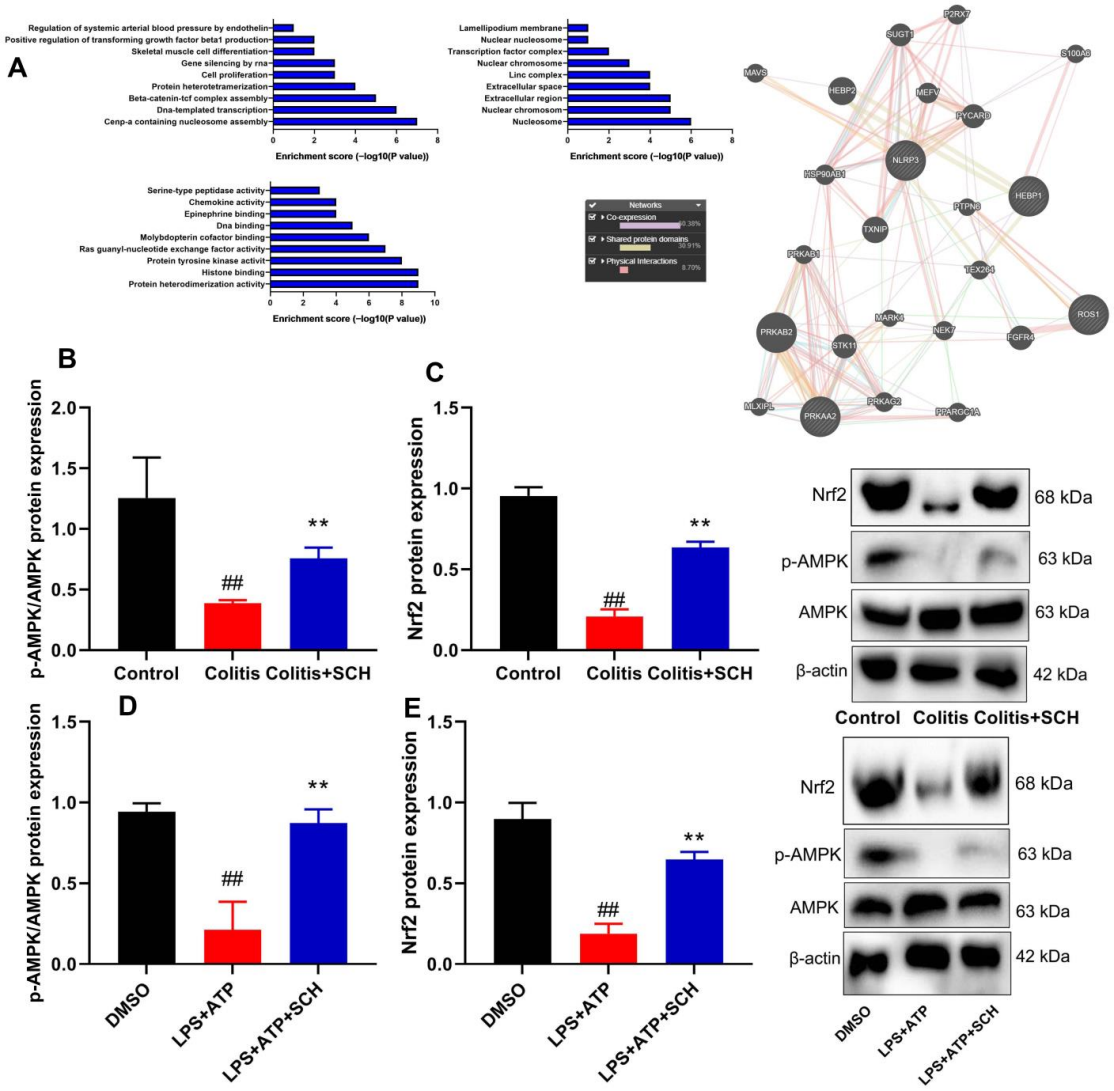
**Systematic understanding of Schisandrin B induced AMPK/Nrf2 signaling pathway *in vivo* and *vitro* model of colitis.** (**A**) Network of potential Schisandrin B targets; (**B**, **C**) p-AMPK and Nrf2 protein expression in mouse colon tissue; (**D**, **E**) p-AMPK and Nrf2 protein expression in intestinal epithelial cells induced by LPS + ATP. ##P<0.01 vs control group; **P<0.01 vs DSS- induced colitis group. Control: blank control group; Colitis: DSS- induced colitis group; Colitis+SCH: DSS- induced colitis mice with Schisandrin. ##P<0.01 vs MDSO group; **P<0.01 vs LPS+ATP induced intestinal epithelial cells group. MDSO: blank control group; LPS+ATP: intestinal epithelial cells with LPS+ATP group; LPS+ATP +SCH: intestinal epithelial cells induced by LPS+ATP with Schisandrin. SCH, Schisandrin B. Data were expressed as mean ± SEM.

TCMSP server (http://ibts.hkbu.edu.hk/LSP/tcmsp.php) was used to further investigate the anti-inflammatory mechanism of Schisandrin B in DSS-induced acute colitis (see physical interactions, pathways and co-localisation in Figure 4A). Obviously, 60.38% showed similar co-expression characteristics, and 30.91% shared the same protein domain. Then, AMPK/Nrf2 signaling for target fishing and pathway was analyzed. Schisandrin B induced p-AMPK and Nrf2 protein expression in colon tissue and intestinal epithelial cells by LPS+ATP (Figure 4B–4E). As shown by these results, Schisandrin B suppressed NLRP3 inflammasome in intestinal epithelial cells by AMPK/Nrf2 signaling.

### Schisandrin B regulated AMPK protein at 316 sites

### The study explored how Schisandrin B induced AMPK protein

**Figure 5:**
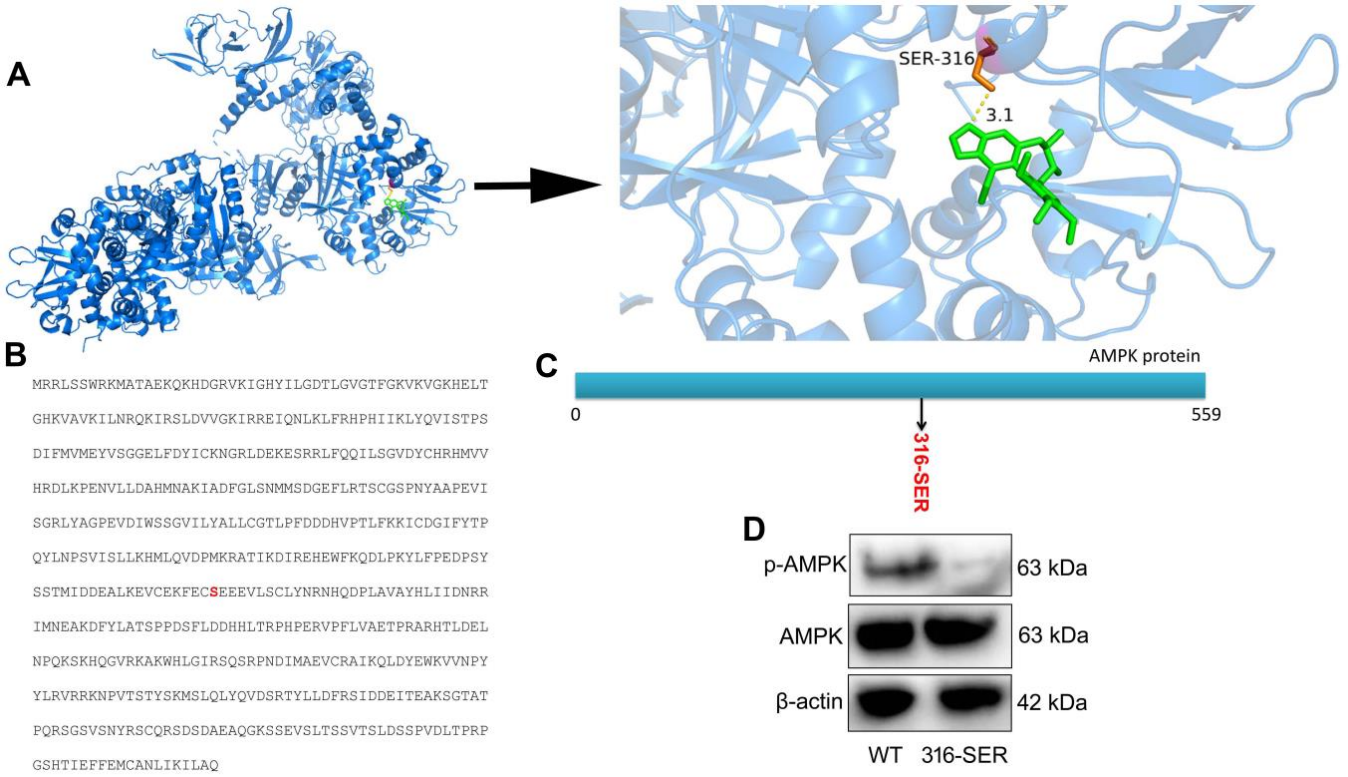
**Schisandrin B regulated AMPK protein at 316 site.** (**A**) protein spatial structure and drug structure; (**B**) amino acid sequence for AMPK protein; (**C**) Schematic diagram of protein mutation; (**D**) p- AMPK protein expression.

According to the degree value and frequency, AMPK corresponding to only one active component in network screening target was selected for molecular docking. Indeed, the binding energy of receptor and ligand is less than - 6.3 kcal / mol, so that it can be considered to have a strong binding activity. Beyond that, the docking results were imported into Python PyMOL 2.5.0 Open Source Software for final visualization and local optimization. The results are displayed in Figure 5A. Furthermore, we conducted protein structure modification and reformed at 316-SER (Serine) of AMPK protein (Figure 5B, 5C). Compared with WT protein expression, p-AMPK protein expression was more significantly suppressed in 316-mutant protein (Figure 5D). Taken together, these results revealed that Schisandrin B combined AMPK protein at 316-SER.

### The inhibition of AMPK reduced the anti-inflammation effects of Schisandrin B on NLRP3 inflammasome

**Figure 6:**
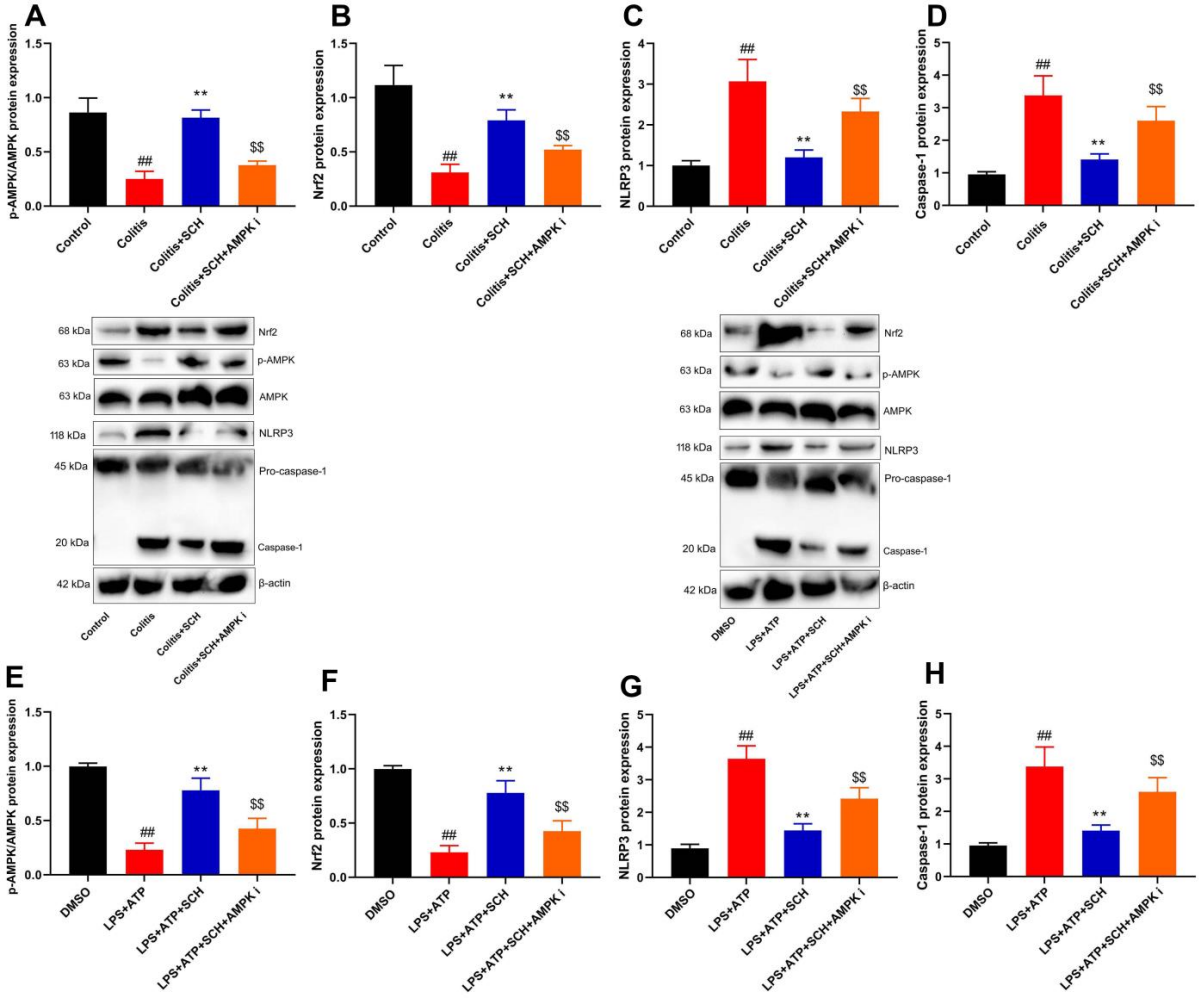
**The inhibition of AMPK reduced the anti-inflammation effects of Schisandrin B on NLRP3 inflammasome.** (**A**–**D**) p-AMPK, Nrf2, NLRP3 and Caspase-1 protein expressions in mouse colon tissue; (**E**–**H**) p-AMPK, Nrf2, NLRP3 and Caspase-1 protein expressions in intestinal epithelial cells induced by LPS + ATP. ##P<0.01 vs control group; **P<0.01 vs DSS- induced colitis group; ^$$^P<0.01 vs Colitis+SCH group. Control: blank control group; Colitis: DSS- induced colitis group; Colitis+SCH: DSS- induced colitis mice with Schisandrin; Colitis+SCH+ AMPK i: DSS- induced colitis mice with Schisandrin and AMPK inhibitor. ##P<0.01 vs MDSO group; **P<0.01 vs LPS+ATP induced intestinal epithelial cells group; ^$$^P<0.01 vs LPS+ATP+SCH group. MDSO: blank control group; LPS+ATP: intestinal epithelial cells with LPS+ATP group; LPS+ATP +SCH: intestinal epithelial cells induced by LPS+ATP with Schisandrin; LPS+ATP+SCH+AMPK i: intestinal epithelial cells induced by LPS+ATP, Schisandrin and AMPK inhibitor. SCH, Schisandrin B. Data were expressed as mean ± SEM.

AMPK inhibitor (Dorsomorphin, 10 mg/kg) not only suppressed p-AMPK and Nrf2 protein expression levels, but also induced NLRP3 and Caspase-1 protein expression levels in DSS-induced colitis mice treated with Schisandrin B, in comparison to the group treated with Schisandrin B (Figure 6A–6D). Next, AMPK inhibitor (Dorsomorphin, 10 μM) suppressed p-AMPK and Nrf2 protein expression levels, and induced NLRP3 and Caspase-1 protein expression levels in intestinal epithelial cells treated with LPS+ATP and Schisandrin B, in comparison to the group treated with Schisandrin B (Figure 6E–6H). As revealed by these results, AMPK was the target spot of Schisandrin B on NLRP3 inflammasome in colitis model.

### Schisandrin B reduced ROS-induced mitochondrial damage

**Figure 7:**
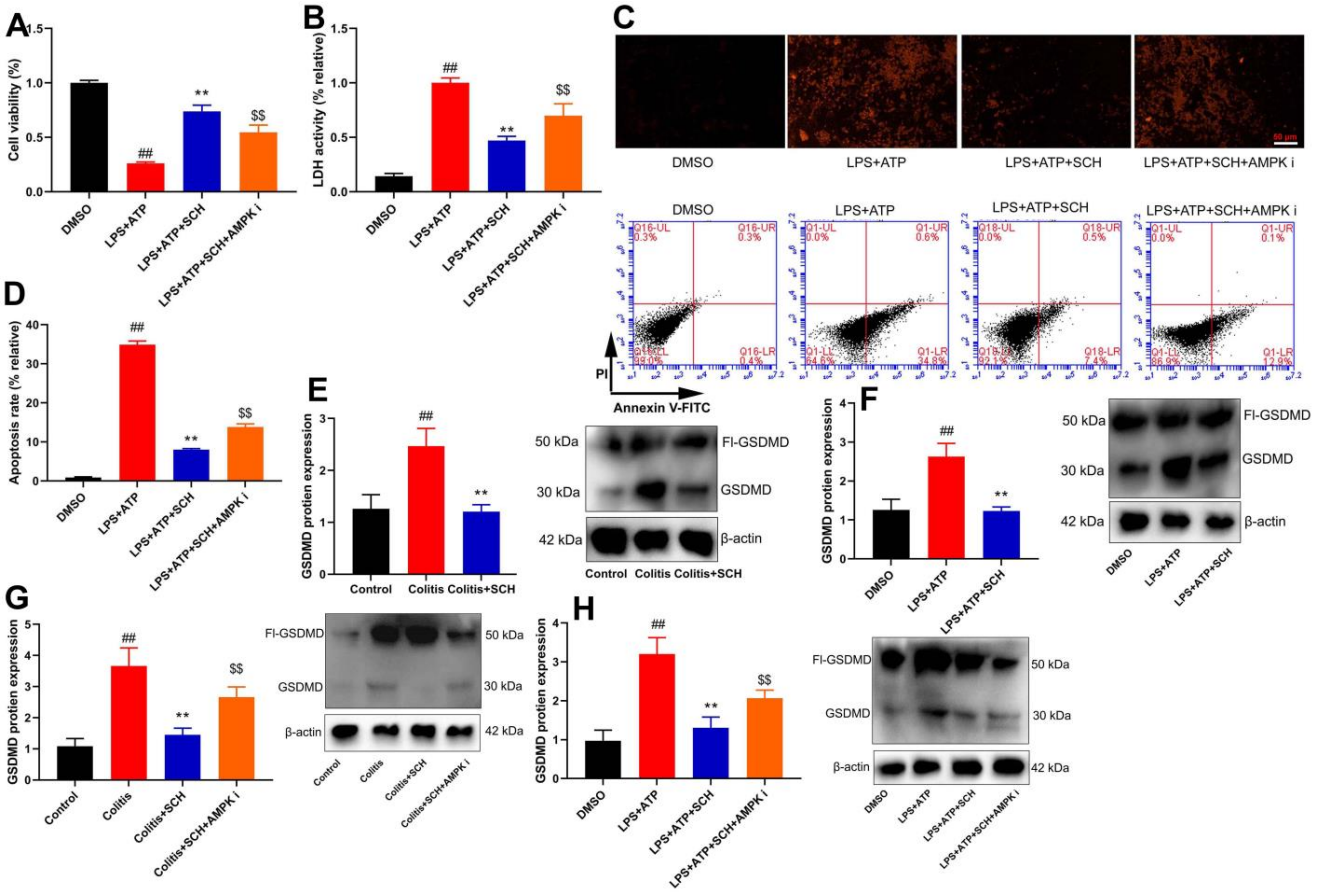
**Schisandrin B reduced epithelial cells injury of colitis through regulating pyroptosis.** (**A**) cell proliferation, (**B**) LDH activity, (**C**) PI staining, (**D**) cell apoptosis rate in epithelial cells induced by LPS + ATP; (**E**) GSDMD protein expression in mouse colon tissue; (**F**) GSDMD protein expression in epithelial cells induced by LPS + ATP; (**G**) GSDMD protein expression in mouse colon tissue by Schisandrin B and AMPK inhibitor; (**H**) GSDMD protein expression in epithelial cells induced by LPS + ATP, Schisandrin B and AMPK inhibitor. ##P<0.01 vs control group; **P<0.01 vs DSS- induced colitis group; ^$$^P<0.01 vs Colitis+SCH group. Control: blank control group; Colitis: DSS- induced colitis group; Colitis+SCH: DSS- induced colitis mice with Schisandrin; Colitis+SCH+ AMPK i: DSS- induced colitis mice with Schisandrin and AMPK inhibitor. ##P<0.01 vs MDSO group; **P<0.01 vs LPS+ATP induced intestinal epithelial cells group; ^$$^P<0.01 vs LPS+ATP+SCH group. MDSO: blank control group; LPS+ATP: intestinal epithelial cells with LPS+ATP group; LPS+ATP +SCH: intestinal epithelial cells induced by LPS+ATP with Schisandrin; LPS+ATP+SCH+AMPK i: intestinal epithelial cells induced by LPS+ATP, Schisandrin and AMPK inhibitor. SCH, Schisandrin B. Data were expressed as mean ± SEM.

**Figure 8:**
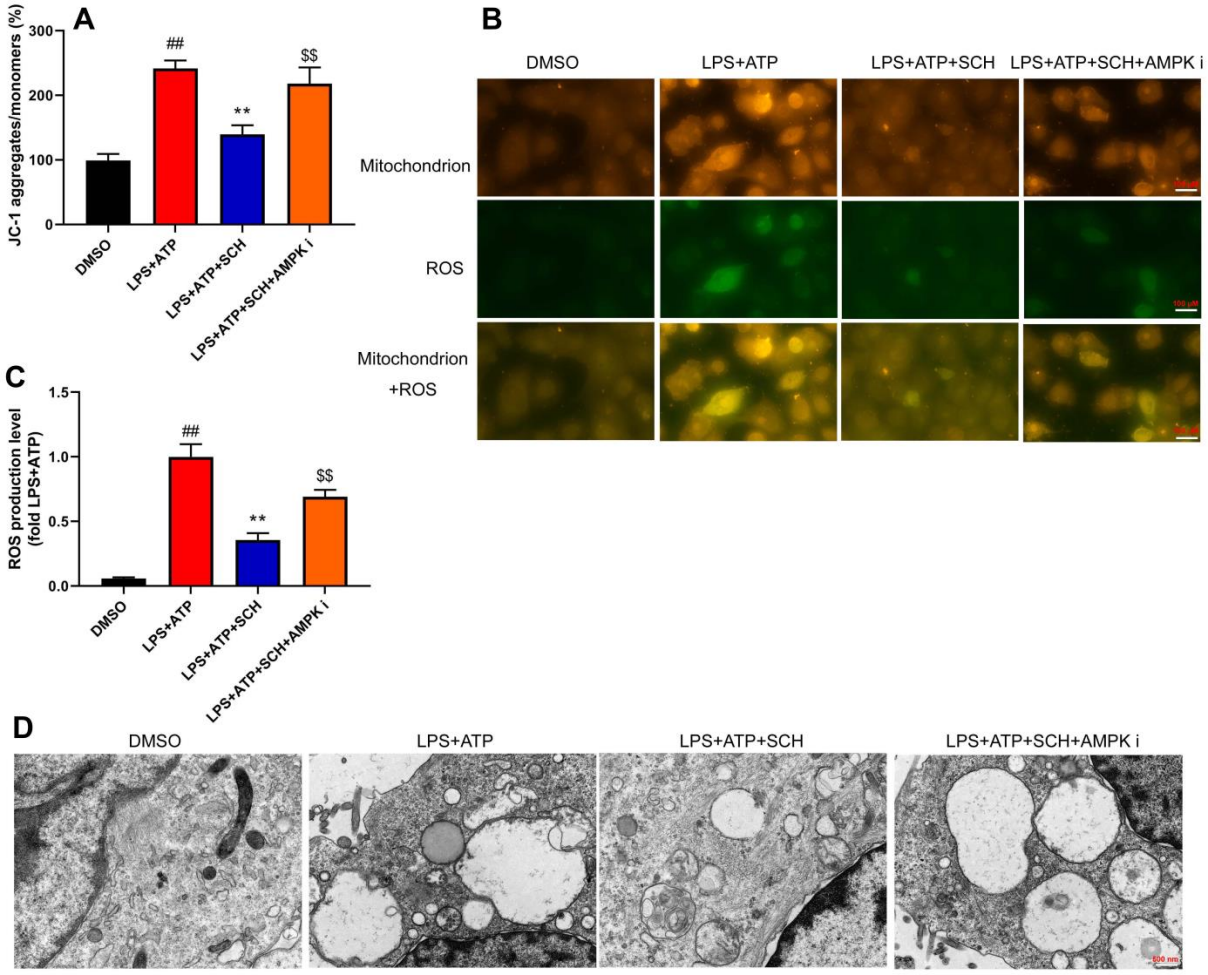
**Schisandrin B reduced ROS-induced mitochondrial damage.** (**A**) Methods: the total number of JC-1 in intestinal epithelial cells induced by LPS + ATP; (**B**) the level of ROS in intestinal epithelial cells induced by LPS + ATP; (**C**) mitochondria and ROS in intestinal epithelial cells by immunofluorescence; (**D**) mitochondria in intestinal epithelial cells by electron microscope. ##P<0.01 vs MDSO group; **P<0.01 vs LPS+ATP induced intestinal epithelial cells group; ^$$^P<0.01 vs LPS+ATP+SCH group. MDSO: blank control group; LPS+ATP: intestinal epithelial cells with LPS+ATP group; LPS+ATP +SCH: intestinal epithelial cells induced by LPS+ATP with Schisandrin; LPS+ATP+SCH+AMPK i: intestinal epithelial cells induced by LPS+ATP, Schisandrin and AMPK inhibitor. SCH, Schisandrin B. Data were expressed as mean ± SEM.

It is believed that ROS-induced mitochondrial damage is a common trigger of various NLRP3-activating agents. Thus, we analyzed whether Schisandrin B suppressed ROS-induced mitochondrial damage in intestinal epithelial cells by LPS+ATP. Obviously, LPS+ATP caused JC-1 aggregates and ROS production levels to increase in intestinal epithelial cells (Figure 7A, 7B). At the same time, Schisandrin B reduced JC-1 aggregates and ROS production levels in intestinal epithelial cells by LPS+ATP (Figure 8A, 8B). AMPK inhibitor (Dorsomorphin, 10 μM) increased JC-1 aggregates and ROS production levels in intestinal epithelial cells by LPS+ATP and Schisandrin B, in comparison to the group treated with Schisandrin B (Figure 7A, 7B). According to immunofluorescence, LPS+ATP enhanced ROS production level (green) and activated mitochondrial damage (red) in intestinal epithelial cells. ROS coincided with mitochondrial damage in intestinal epithelial cells (Figure 8C). In addition, Schisandrin B inhibited ROS production level (green) and reduced mitochondrial damage (red) in intestinal epithelial cells by LPS+ATP (Figure 8C). AMPK inhibitor (Dorsomorphin, 10 μM) promoted ROS production level (green) and mitochondrial damage (red) in intestinal epithelial cells by LPS+ATP and Schisandrin B, in comparison to the group treated with Schisandrin B (Figure 8C). More importantly, electron microscope indicated that LPS+ATP induced mitochondrial damage in intestinal epithelial cells (Figure 8D). Schisandrin B restored mitochondrial damage in intestinal epithelial cells by LPS+ATP (Figure 8D). AMPK inhibitor (Dorsomorphin, 10 μM) amplified mitochondrial damage in intestinal epithelial cells LPS+ATP and Schisandrin B, in comparison to the group treated with Schisandrin B (Figure 8D). Taken together, these results indicated that ROS-induced mitochondrial damage might be a dominant mechanism of underlying activation of the NLRP3 inflammasome in colitis by treatment with Schisandrin B through AMPK/Nrf2 signaling pathway.

### Schisandrin B reduced epithelial cells damage of colitis through regulating pyroptosis

Pyroptosis is an important factor for intestinal cell damage or intestinal cell deaths to lead the occurrence and development of diseases of colitis. Cell viability was reduced, and LDH activity level and cell apoptosis were increased in intestinal epithelial cells by LPS+ATP (Figure 7A–7D). Schisandrin B not only increased cell viability but also reduced LDH activity level and cell apoptosis in intestinal epithelial cells by LPS+ATP (Figure 7A–7D). AMPK inhibitor (Dorsomorphin, 10 μM) decreased cell viability and increased LDH activity level and cell apoptosis in intestinal epithelial cells by LPS+ATP and Schisandrin B, in comparison to the group treated with Schisandrin B (Figure 7A–7D). Figure 7D showed that AMPK inhibitor (Dorsomorphin, 10 μM) induced early apoptosis of intestinal epithelial cells by LPS+ATP and Schisandrin B, in comparison to the group treated with Schisandrin B. GSDMD protein expression was induced in intestinal epithelial cells by LPS+ATP (Figure 7E), and Schisandrin B suppressed GSDMD protein expression in intestinal epithelial cells by LPS+ATP (Figure 7E). GSDMD protein expression in colon tissue of DSS induced colitis mice model is shown in Figure 7F. Administration of Schisandrin B suppressed GSDMD protein expression in colon tissue of DSS induced colitis mice model (Figure 7F). AMPK inhibitor (Dorsomorphin, 10 mg/kg) induced GSDMD protein expression in colon tissue of colitis mice treated with Schisandrin B more significantly in comparison to Schisandrin B group (Figure 7G). AMPK inhibitor (Dorsomorphin, 10 μM) induced GSDMD protein expression in intestinal epithelial cells treated by LPS+ATP and Schisandrin B, in comparison to Schisandrin B group (Figure 7H). Collectively, these data indicated that Schisandrin B reduced epithelial cells damage of colitis through regulating pyroptosis via AMPK/Nrf2 signaling pathway.

## DISCUSSION

UC is a chronic inflammatory disease with increasing incidence and prevalence in many countries [36]. It has been determined that the onset of UC is associated with multiple genetic and environmental factors [37]. Recent evidence indicates that intestinal permeability plays a vital role in host defense [38]. UC patients show increased intestinal mucosal permeability, which is associated with disease severity [39]. The disorder of the intestinal epithelial barrier plays an important role in the inflammatory process [40]. Therefore, it is necessary to clarify the potential mechanism of intestinal epithelial barrier damage induced by UC inflammation to identity novel therapeutic approaches [41]. In this study, we found that Schisandrin B presented colitis and reduced inflammation *in vivo* and *vitro* model of colitis. Li et al. revealed Schisandrin B prevents ulcerative colitis via gut microbiota in an *in vivo* and *in vitro* model [42]. Importantly, we identified that Schisandrin B may be a possible drug for the treatment of colitis.

UC is an autoimmune disease caused by excessive activation of the immune system. DSS-induced colitis is commonly used as the animal model for UC [43]. DSS can destroy the integrity of the intestinal epithelial barrier, increase epithelial permeability and cause the entrance of intestinal mucosal antigens and microorganisms into the mucosa, leading to the infiltration of immune cells (macrophages and neutrophils) in the lamina propria and submucosa [44, 45]. In addition, DSS can cause intense inflammation and the release of many pro-inflammatory cytokines [44]. We found that Schisandrin B suppressed NLRP3 inflammasone *in vivo* and *vitro* model of colitis. Chen et al. showed that Schisandrin B attenuates inflammation in asthma by inhibiting NLRP3 inflammasome activation [42]. Thus, our findings showed that NLRP3 inflammasone, a target spot of Schisandrin B on colitis to reduce inflammation factor of intestinal epithelial cells.

AMPK is mainly involved in regulating sugar, lipid and energy metabolism in the body [46]. Studies have demonstrated that AMPK activation can inhibit inflammation and oxidative stress [47, 48]. Nrf2 is a key molecule for the transcriptional regulation of antioxidant factors in the body [49]. The Nrf2 /HO-1 signaling pathway plays an important role in maintaining the antioxidant response of the body. Moreover, multiple studies have revealed that Nrf2 can be activated by AMPK [50]. The Nrf2 system can interact with NLRP3 inflammasome [51]. Moreover, the Nrf2 system can reduced the expression of downstream genes of the NLRP3 inflammasome, and the activation of NLRP3 inflammasome can also regulate the Nrf2 system [52]. The interaction between Nrf2 system and NLRP3 inflammasome is associated with acute and chronic inflammation, oxidative stress and autophagy [53]. In this study, we identified that Schisandrin B induced AMPK/Nrf2 signaling pathway and reduced ROS-induced mitochondrial damage in model of colitis. The inhibition of AMPK reduced the anti-inflammation effects of Schisandrin B on NLRP3 inflammasome. Zhao et al. suggested that Schisandrin B attenuates hypoxia/reoxygenation injury by activating the AMPK/Nrf2 signaling pathway [54]. Thus, Schisandrin B induced AMPK/Nrf2 signaling pathway to suppress NLRP3 inflammasome in model of colitis by the inhibition of ROS-induced mitochondrial damage.

NLRP3 is expressed by a variety of cells, mainly hematopoietic cells [17]. However, the expression of NLRP3 is also detected in glandular epithelial structures of the internal layer including the small intestine, stomach, and colon [17]. Pyroptosis is a novel type of pro-inflammatory programmed cell death, which is different from necrosis and apoptosis and is closely associated with the activation of NLRP3 inflammasome [11]. After stimulation, NLRP3 binds to ASC to recruit Caspase-1 to form an inflammasome complex [20]. Subsequently, it is cleaved to form active IL-1β. Caspase-1 also cleaves Gasdermin D to generate N-terminal fragments (GSDMD-N) and forms membrane pores to trigger the release of IL-1β [18]. Accumulative evidence suggests that the activation of NLRP3 inflammasomes and pyroptosis are associated with the inflammatory process of colitis [55]. Moreover, we also noticed that Schisandrin B reduced epithelial cells injury of colitis through regulating pyroptosis by AMPK/Nrf2/NLRP3 inflammasome. Guo et al. showed that Schisandrin B suppressed NLRP3 inflammasome activation-mediated IL-1β level and pyroptosis [30]. Thus, we conclude that Schisandrin B suppressed NLRP3 inflammasome activation-mediated IL-1β level and pyroptosis in intestinal epithelial cells of colitis model by the activation of AMPK/Nrf2 dependent signaling- ROS-induced mitochondrial damage.

**Figure 9:**
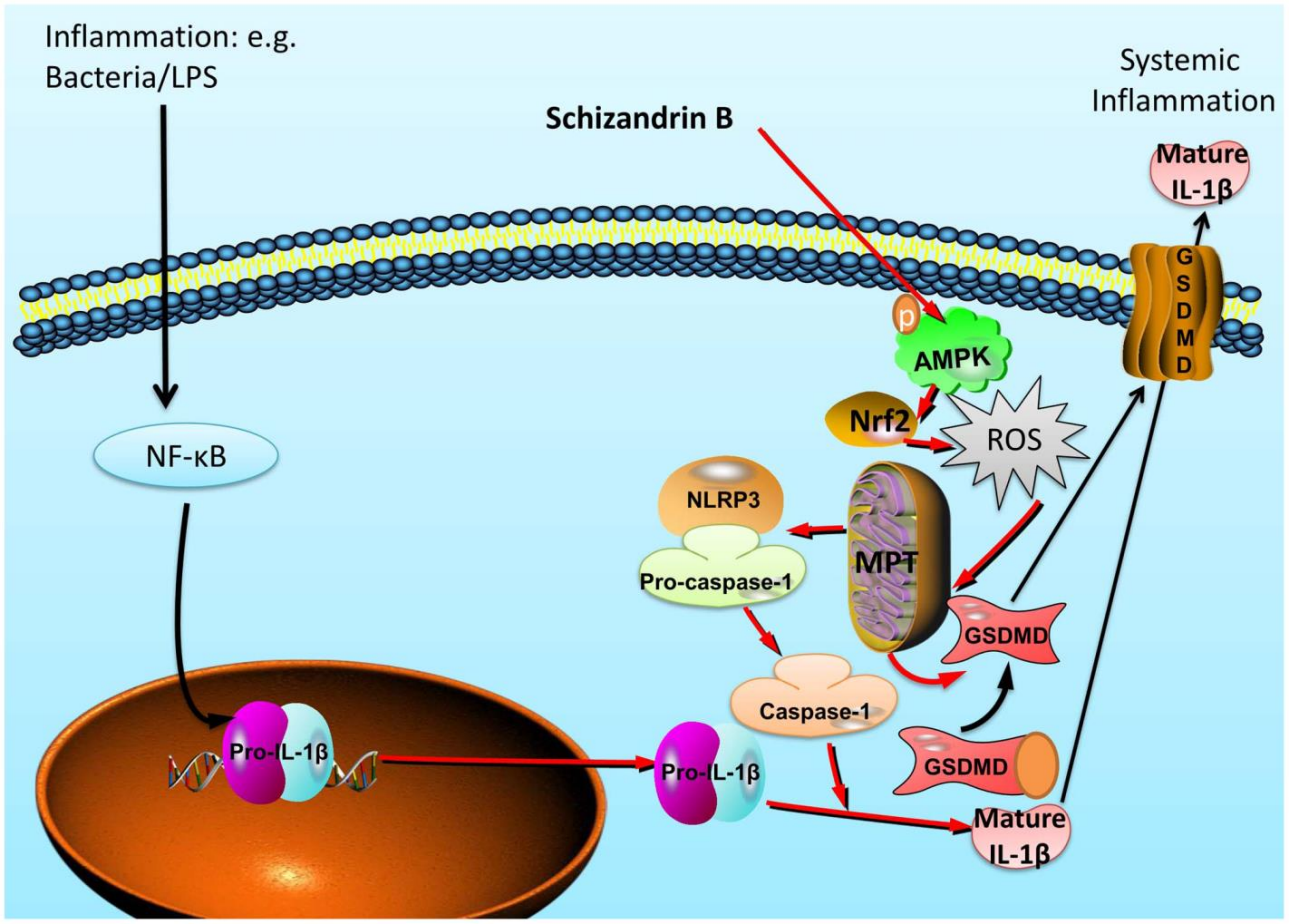
Schisandrin B reduces the epithelial cells injury of colitis through regulating pyroptosis by AMPK/Nrf2/NLRP3 inflammasome.

In summary, our findings for first time indicated that Schisandrin B reduces the epithelial cells injury of colitis through regulating pyroptosis by AMPK/Nrf2/NLRP3 inflammasome (Figure 9). Our study also provides evidence that Schisandrin B may be of significant therapeutic approach in the treatment of acute colitis.
